# Tumor-associated tissue eosinophilia predicts favorable clinical outcome in solid tumors: a meta-analysis

**DOI:** 10.1186/s12885-020-06966-3

**Published:** 2020-05-20

**Authors:** Guoming Hu, Shimin Wang, Kefang Zhong, Feng Xu, Liming Huang, Wei Chen, Pu Cheng

**Affiliations:** 1 Department of General Surgery (Breast and Thyroid Surgery), Shaoxing People’s Hospital (Shaoxing Hospital, Zhejiang University School of Medicine), Zhejiang, 312000 China; 2 Department of Nephrology, Shaoxing People’s Hospital (Shaoxing Hospital, Zhejiang University School of Medicine), Zhejiang, 312000 China; 3 Department of Gynecology, Second Affiliated Hospital, Zhejiang University School of Medicine, Zhejiang University, Hangzhou, 310009 China

**Keywords:** Tumor-associated tissue eosinophilia, Favorable outcome, Human solid tumor, Meta-analysis

## Abstract

**Background:**

Activated eosinophils have been deemed to affect carcinogenesis and tumor progression via various mechanisms in tumor microenvironment. However, the prognostic role of tumor-associated tissue eosinophilia (TATE) in human cancers remains controversial. Therefore, we conducted this meta-analysis to better comprehend the association between TATE and clinical outcomes of patients.

**Methods:**

We searched PubMed, Embase and EBSCO to determine the researches assessing the association between TATE and overall survival (OS) and/or disease-free survival (DFS) in patients with cancer, then combined relevant data into hazard ratios (HRs) or odds ratio (OR) for OS, DFS and clinicopathological features including lymph node metastasis etc. with STATA 12.0.

**Results:**

Twenty six researches with 6384 patients were included in this meta-analysis. We found that the presence of TATE was significantly associated with improved OS, but not with DFS in all types of cancers. In stratified analyses based on cancer types, pooled results manifested that the infiltration of eosinophils was remarkably associated with better OS in esophageal carcinoma and colorectal cancer. In addition, TATE significantly inversely correlated with lymph node metastasis, tumor stage and lymphatic invasion of cancer.

**Conclusion:**

TATE promotes survival in cancer patients, suggesting that it is a valuable prognostic biomarker and clinical application of biological response modifiers or agonists promoting TATE may be the novel therapeutic strategy for patients.

## Background

Tumor microenvironment (TME) linked closely with the initiation, promotion, and progression of cancer [1]. Innate and adaptive immunocytes such as mast cells, macrophages and memory T lymphocytes etc. are the vital components of TME [2]. Multitudinous studies have demonstrated that these immune cells were significantly associated with survival in solid tumors [3, 4]. However, it is essential to distinguish among different types of immune cells as they may play differential roles in the TME. Eosinophils, as the important component of innate immune cells, have proven to play significant roles in a multitude of solid tumors.

Eosinophils are granulocytic leukocytes that are associated with multitudinous pathologic conditions including allergic reactions, parasitic and bacterial infections etc. [5] These cells secrete massive proteins and cytokines upon activation and are involved in a variety of other functions including inducing tissue remodeling and promoting antigen presentation [6]. In the last decade, activated eosinophils have been deemed to affect carcinogenesis and tumor progression via various mechanisms including modulating innate and adaptive immune responses in TME [7]. Eosinophils infiltrating into tumor is also called tumor-associated tissue eosinophilia (TATE) [8]. Recent researches have investigated the TATE in tumor progression and survival, but their results were inconsistent even contradictory [9]. Hence, it needs further evaluation. In addition, the potential of TATE as prognostic biomarker and therapeutic strategy is also required to be investigated.

Herein, we carried out this meta-analysis to expound the relation between TATE and clinical outcomes including overall survival (OS) and disease-free survival (DFS) in patients with cancer.

## Methods

### Search strategy

This meta-analysis was guided by the PRISMA (Preferred Reporting Items for Systematic Reviews and Meta-Analysis) Statement issued in 2009 (Checklist S1). PubMed, Embase and EBSCO were searched for researches from 1980 to May 15th 2019. The keywords applied for search were: (eosinophil [Title/Abstract] OR eosinophilia [Title/Abstract]) AND (neoplasms [Title/Abstract] OR tumor [Title/Abstract] OR cancer [Title/Abstract] OR carcinoma [Title/Abstract]).

### Inclusion and exclusion criteria

Researches included in this meta-analysis should meet the following inclusion criteria: (1) been published as original articles; (2) investigated human subjects; (3) examined eosinophils in primary tumor tissues; (4) reported hazard ratios (HRs) with 95% confidence interval (CI), or Kaplan – Meier curves of eosinophil infiltration with clinical outcomes.

The exclusion criteria were as follows: researches (1) were not published as research articles or full texts including commentaries, case reports, letters to the editors and meeting abstracts; (2) didn’t offer ample data to obtain HRs; (3) investigated eosinophils in metastases or not in tumor tissues.

### Endpoints

In this study, OS and DFS were regarded as the primary and second endpoint respectively.

### Data extraction

GM.H. and SM.W. reviewed and recorded data including number of patients, method to quantify eosinophils, cutoff value to determine TATE and time of follow-up etc. independently. OS, DFS and clinicopathological features such as tumor, node, metastasis (TNM) stage and lymphatic invasion were extracted from the text, tables, or Kaplan – Meier curves.

### Quality assessment

Two authors independently assessed the quality of included cohort researches with Newcastle–Ottawa Scale (NOS), [10] and achieved consensus for each item under the help of third or more authors. Research scored 6 or above was regarded as high quality.

### Statistical analysis

We combined extracted data using STATA 12.0 analysis software, and estimated statistical heterogeneity with the chi-squared based Q-test or *I*^*2*^ (25% was considered low-level heterogeneity, 25–50% moderate-level heterogeneity, and 50% high-level heterogeneity) [11]. Data were pooled based on the random-effect model in the presence of heterogeneity, [12] otherwise, the fixed-effect model was applied [13]. In addition, stratified analyses were conducted based on tumor types; sensitivity analysis, Begg’s funnel plot and Egger’s test [14] were employed to explore the impact of each research on the overall result and potential publication bias respectively. All *P* values were two-sided and below 0.05 was treated as statistical significance.

## Results

### Search results and description of studies

Flow chart diagram of research selection was displayed in Fig. S1. Twenty six researches with 6384 patients were ultimately included in this meta-analysis [15–40]. And all the researches were scored 6 or above after careful evaluation with the Newcastle–Ottawa Scale (NOS); Characteristics of those researches being in the light of the inclusion criteria and suitable for data incorporation were exhibited in Table 1 and Table S1.

**Table 1:** Main characteristics of the included studies

Study	Year	Tumor type	No. of Patients	Male/Female	median age (range) (year)	Staining	TATE: Present / absent	Tumor stage	median follow-up date (months)	Survival	Quality Score (NOS)
Peurala, E. etal [15]	2018	Oral cancer	99	55/44	65.3	H&E	51/47	I - III	40.7	OS	8
Oliveira, D. T. etal [16]	2012	Oral cancer	71	55/16	59 (35, 77)	H&E	35/36	I - II	NR	DFS	7
Tostes Oliveira, D. etal [19]	2009	Oral cancer	43	27/16	55.79 (28, 83)	H&E	21/22	I - IV	(3, 229)	OS	7
Dorta, R. G. etal [17]	2002	Oral cancer	125	105/20	58 (30, 95)	H&E	57/68	II - III	88.2 (0, 287.4)	OS, DFS	7
Dante, P. etal [40]	2019	Tongue Carcinoma	259	223/36	53.0 ± 12.2	H&E	NR	I - IV	NR	OS, DFS	8
Alrawi, S. J. etal [18]	2005	Head and neck carcinoma	87	NR	(41, 76)	H&E	13/7	II - IV	36 (6, 216)	OS, DFS	7
Ercan, I. etal [20]	2005	Laryngeal carcinoma	78	78/0	55.9 (35, 80)	H&E	25/53	NR	41.91	OS	7
Sassler, A. M. etal [21]	1995	Laryngeal carcinoma	248	NR	NR	H&E	56/192	III - IV	48	OS, DFS	6
Thompson, A. C. etal [22]	1994	Laryngeal carcinoma	104	85/19	64.6 (39, 91)	H&E	31/73	NR	≥ 60	OS	6
Fujii, M. etal [23]	2002	Nasopharyngeal carcinoma	53	40/13	49.4 (15, 81)	H&E	26/27	I - IV	90.5 (35.3, 199.9)	DFS	7
Leighton, S. E. etal [24]	1996	Nasopharyngeal carcinoma	96	68/28	NR	H&E	65/31	NR	57	OS, DFS	6
Harbaum, L. etal [25]	2015	Colorectal cancer	381	166/215	68.5	H&E	101/280	I - IV	45 (1, 182)	OS	8
Fernandez-Acenero, M. J. etal [26]	2000	Colorectal cancer	126	70/56	67.35 (32, 87)	H&E	29/97	Duke’s A-C	≥ 60	OS, DFS	8
Nielsen, H.J. etal [27]	1999	Colorectal cancer	584	240/344	61 (49, 75)	H&E	150/115	Duke’s A-D	61 (49, 75)	OS	7
Prizment, A. E etal [28]	2016	Colorectal cancer	441	0/441	(55, 69)	H&E; EPX	197 /244	NR	60	OS	8
Zhang, Y. etal [29]	2014	Esophageal carcinoma	36	25/11	59 (45, 77)	H&E	18/18	I - IV	22 (2, 143)	OS	7
Ishibashi, S. etal [30]	2006	Esophageal carcinoma	97	82/15	62.7 ± 8.9	H&E	30/31	NR	61.7 (5.3, 165.4)	OS	7
Hollander, P. etal [31]	2018	Hodgkin’s lymphoma	459	242/217	< 45: 68%; ≥45: 32%	H&E	NR	I - IV	154.8	OS	8
Kereszres, K. etal [32]	2007	Hodgkin’s lymphoma	104	54/50	33 (12, 72)	H&E	64/40	I - IV	110 (24, 214)	OS, DFS	7
von Wasielewski, R. etal [33]	2000	Hodgkin’s lymphoma	1511	745/766	(15, 75)	H&E	510/823	I - IV	120	OS	8
Enblad, G.etal [34]	1993	Hodgkin’s lymphoma	140	NR	45 (11, 94)	H&E	26/114	I - IV	48 (20, 85)	DFS	6
van Driel, W.J. etal [35]	1996	Cervical cancer	83	0/83	42.1	H&E	NR	I - IIA	44.6 (5, 108)	OS, DFS	7
Bethwaite, P. B. etal [36]	1993	Cervical cancer	67	0/67	43.7 (25, 76)	H&E	28/39	IB	62.4 (1, 93)	OS	7
Flamm, J. etal [37]	1992	Bladder cancer	428	289/139	70.2 (29, 91)	H&E	99/329	NR	84	OS	7
Iwasaki, K. etal [38]	1986	Gastric cancer	647	364/283	(22, 84)	H&E	157/490	I - IV	(8, 92)	OS	7
Ono, Y. etal [39]	2002	Penile cancer	17	17/0	68 (36, 84)	H&E	9/8	I - IV	NR	OS	6

*H&E* haematoxilyn and eosin, *EPX* eosinophil peroxide, *NR *not reported

### Meta-analyses

#### Overall survival (OS)

**Fig. 1:**
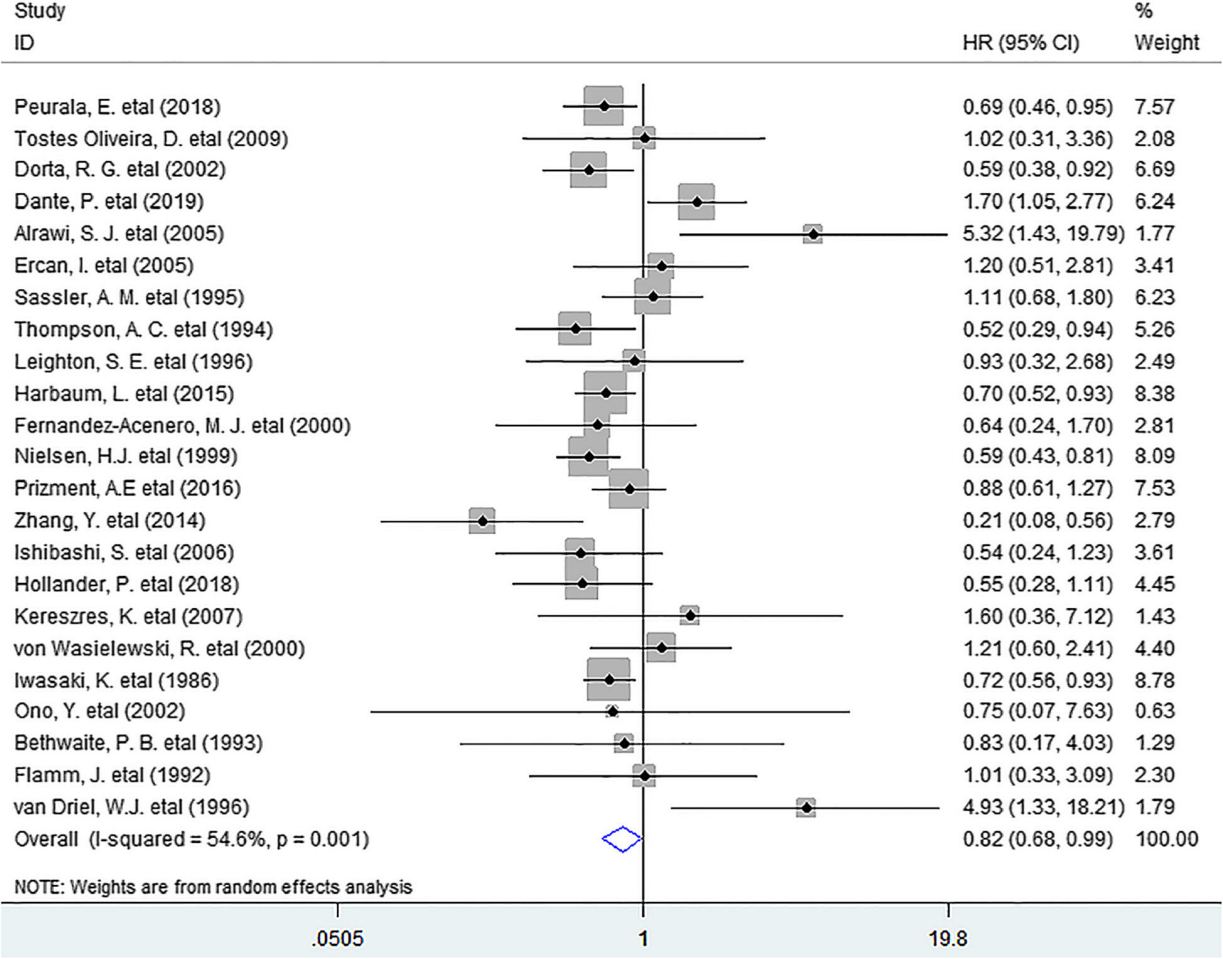
Forest plots describing HR of the association between TATE and OS in human solid tumors

In this meta-analysis, we discovered that the presence of TATE was notably associated with improved OS (HR = 0.82, 95% CI 0.68 to 0.99, *P* = 0.041) in patients with solid tumor. (Fig. 1).

**Fig. 2:**
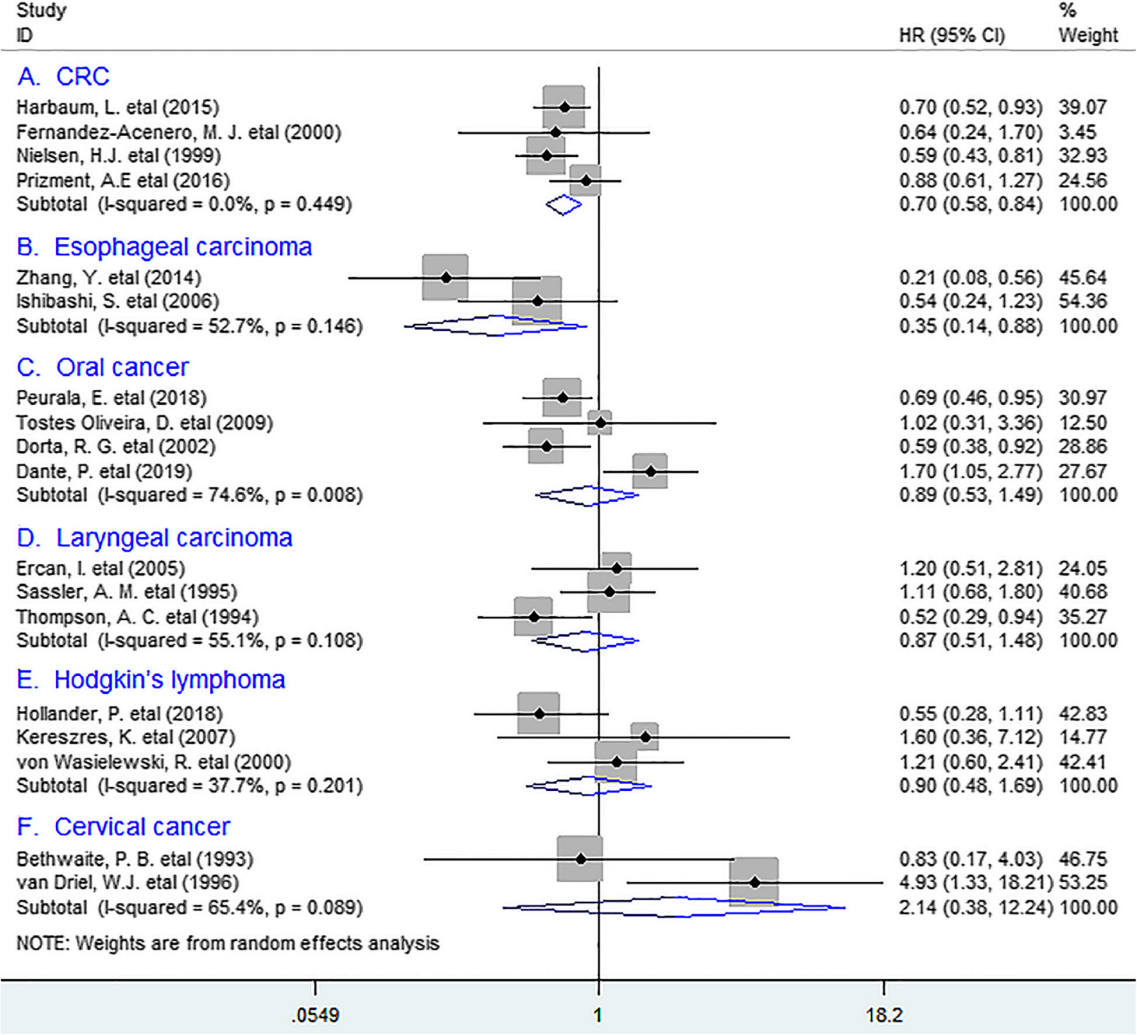
Stratified analyses describing HRs of the association between TATE and OS

In stratified analyses according to tumor types, the combined results manifested that TATE was markedly associated with better OS in colorectal cancer (CRC) (HR = 0.70, 95% CI 0.58 to 0.84, *P* = 0.000), with no heterogeneity detected (*I*^*2*^ *= 0%, P = 0.449)*. Similar data was obtained between TATE and OS in esophageal carcinoma (EC) (HR = 0.35, 95% CI 0.14 to 0.88, *P* = 0.026); Whereas no distinct relation existed between eosinophil infiltration and OS in oral cancer (OC) (HR = 0.89, 95% CI 0.53 to 1.49, *P* = 0.657), laryngeal carcinoma (HR = 0.87, 95% CI 0.51 to 1.48, *P* = 0.599), Hodgkin’s lymphoma (HR = 0.90, 95% CI 0.48 to 1.69, *P* = 0.741) or cervical cancer (HR = 2.14, 95% CI 0.38 to 12.24, *P* = 0.391). (Fig. 2).

#### Disease-free survival (DFS)

**Fig. 3:**
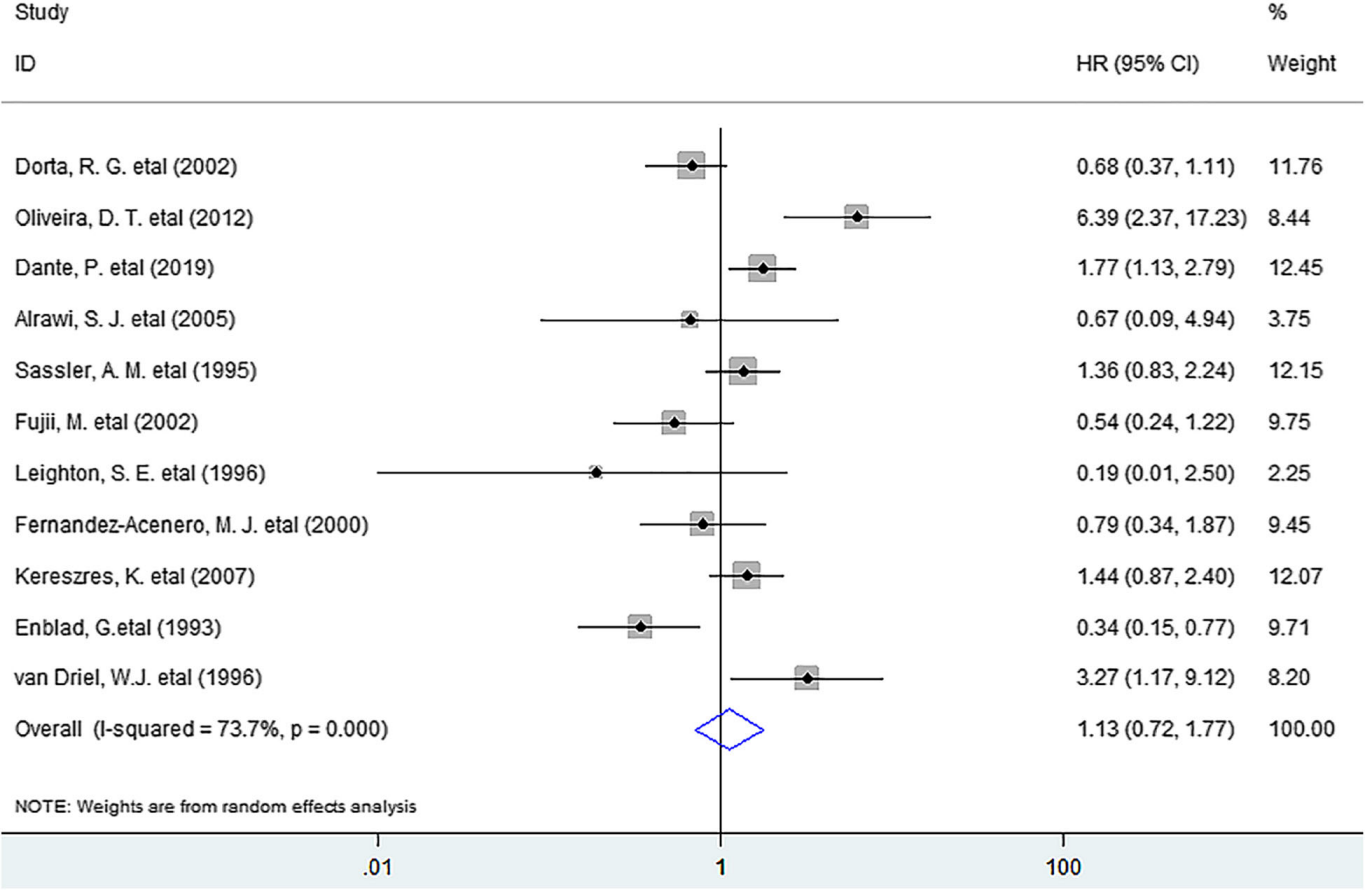
Forest plots describing HR of the association between TATE and DFS in human solid tumors

**Fig. 4:**
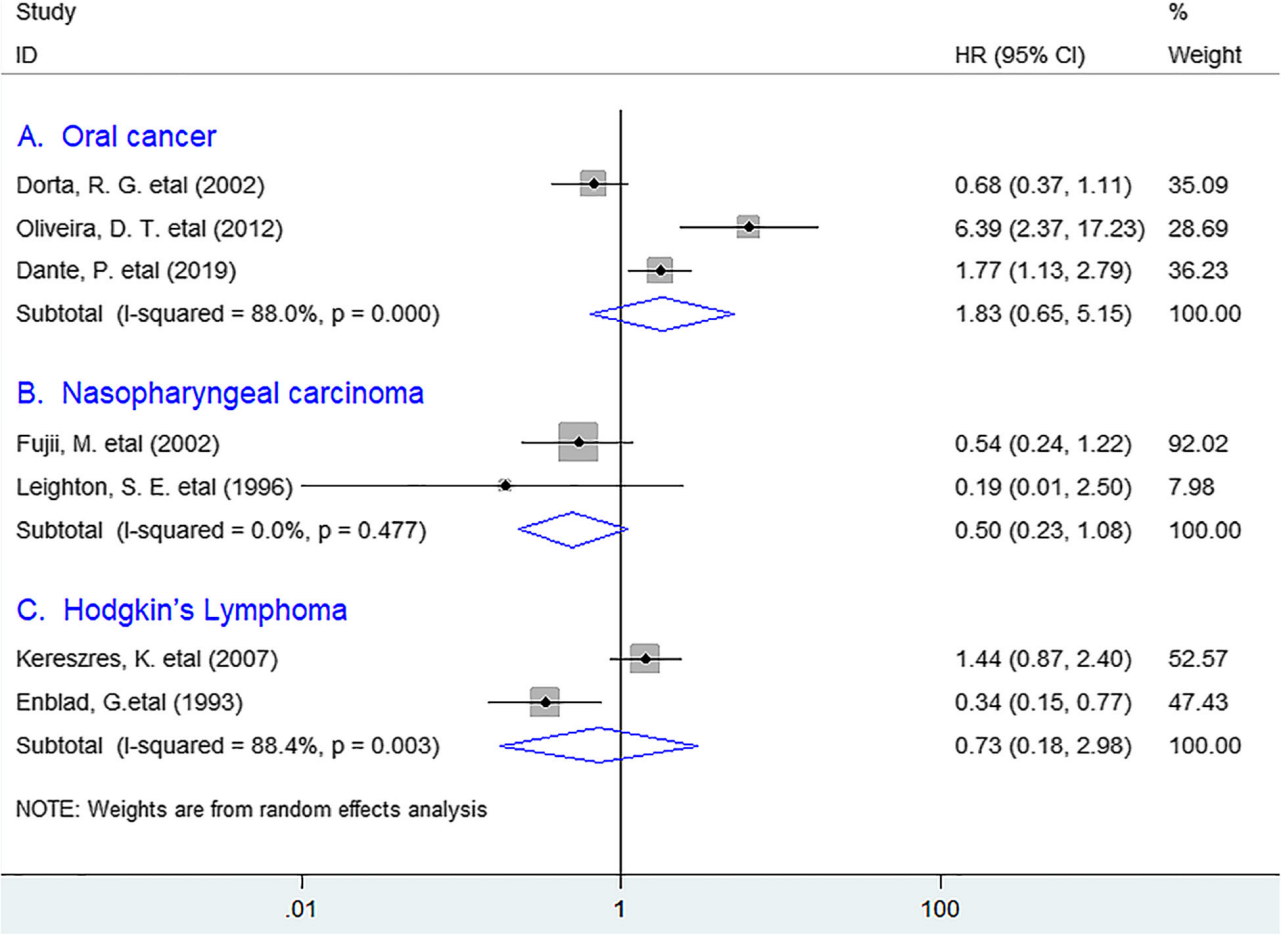
Stratified analyses describing HRs of the association between eosinophil infiltration and DFS

As for DFS, the meta-analysis indicated that no noticeable association existed between eosinophil infiltration and DFS (HR = 1.13, 95% CI 0.72 to 1.77, *P* = 0.598) in solid tumors. (Fig. 3) In the stratified analyses, the incorporated results revealed that TATE was not significantly associated with improved DFS in oral cancer (HR = 1.83, 95% CI 0.65 to 5.15, *P* = 0.253), nasopharyngeal carcinoma (HR = 0,50, 95% CI 0.23 to 1.08, *P* = 0.079) or Hodgkin’s lymphoma (HR = 0.73, 95% CI 0.18 to 2.98, *P* = 0.657). (Fig. 4).

#### Clinicopathological features

**Fig. 5:**
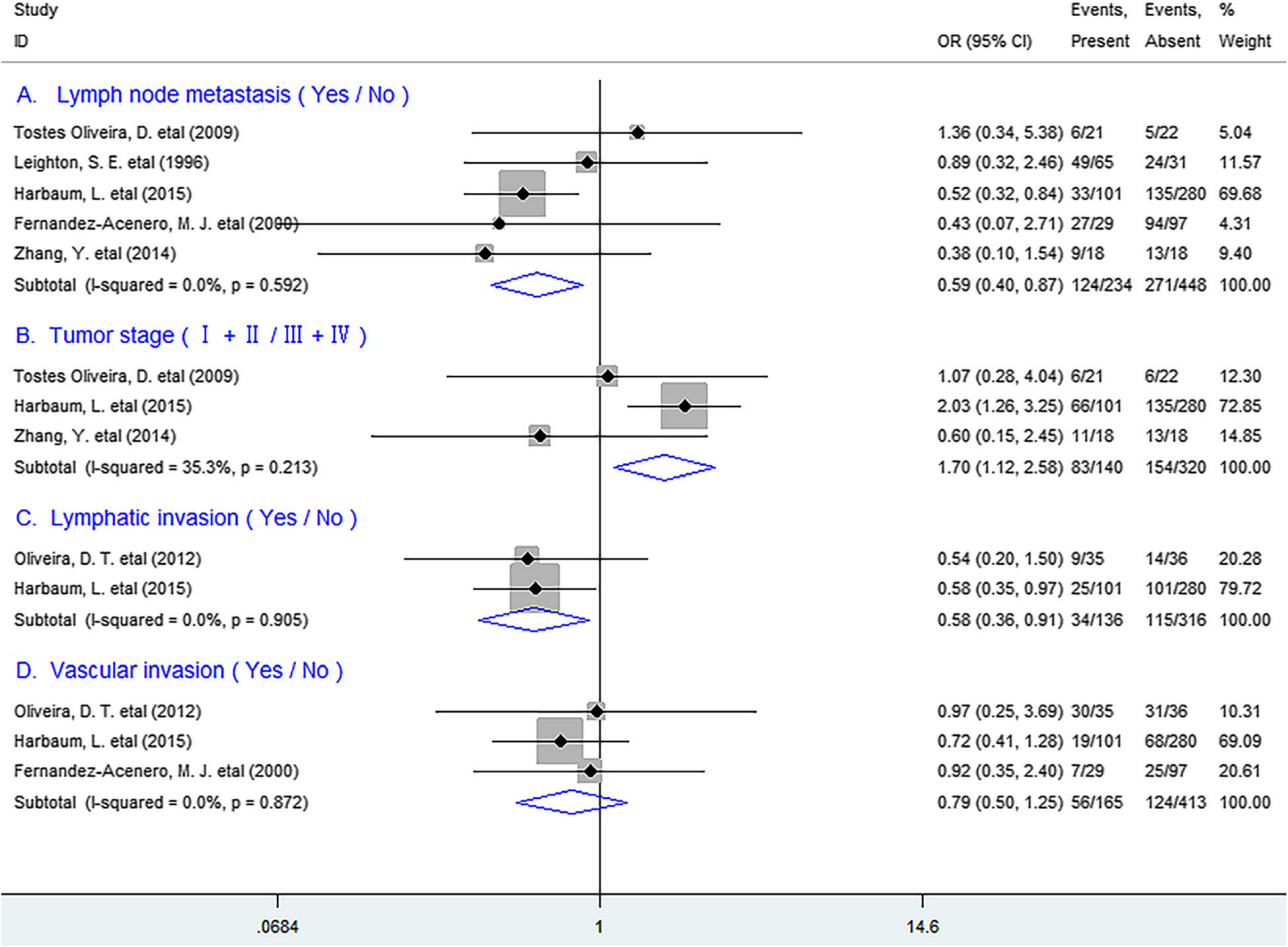
Forest plots indicating ORs of the association between eosinophil infiltration and clinicopathological feature

We next tested the relation between TATE and clinicopathological features, and found that TATE was remarkably inversely correlated with lymph node metastasis (OR = 0.59, 95% CI 0.40 to 0.87, *P* = 0.007), TNM stage (OR = 1.70, 95% CI 1.12 to 2.58, *P* = 0.013) and lymphatic invasion (OR = 0.58, 95% CI 0.36 to 0.91, *P* = 0.018), but not with vascular invasion (OR = 0.79, 95% CI 0.50 to 1.25, *P* = 0.308) of patients. (Fig. 5).

#### Sensitivity analysis

Sensitivity analysis demonstrated that each included research had no impact on the overall result for OS or DFS. (Fig. S2).

#### Publication bias

No publication bias existed between TATE and OS (*P* = 0.152) or DFS (*P* = 0.876) in patients by Funnel plot (Fig. S3) and Egger’s test.

## Discussion

Eosinophilia is commonly associated with allergies, helminth infections and several inflammatory states. Recently, it has also been noted in human solid tumors. The present meta-analysis revealed that TATE had a positive effect in improving survival in human solid tumors, especially in CRC and EC. Moreover, It significantly inversely correlated with lymph node metastasis etc. of tumor. Hence, these data offered important evidence in uncovering the positive prognostic role of TATE in human solid tumors.

The close relation between TATE and better clinical outcome identified in this study possibly attribute to the following reasons: eosinophils in the TME can express same receptors and mediators such as granzyme A etc. as cytotoxic T lymphocytes (CTLs) and be directly involved in anti-tumor response, [41] and they can also secret several chemokines including CCL5, CXCL9 to promote anti-tumor immunity through attracting CD8^+^ T cells to the tumor site [42]. In addition, eosinophils are capable of regulating immunity, for instance, they can release major basic protein (MBP), a highly cationic protein to stimulate maturation of dendritic cells by increasing cell surface activation markers including MHC-II, CD80 and CD86, [43] which has the potential to overcome immune tolerance and induce anti-tumor immunity with the powerful antigen-presentation ability [44]. Furthermore, they can induce cell death of various cell lines such as colo-205 cell line with some selectivity in their tumoricidal properties, which are dependent on the CD11a/CD18-mediated stable contacts with target cells [45]. Hence, it is rational to conclude that TATE is capable of regulating tissue homeostasis of the TME and inhibiting tumor growth and metastasis thereby improving survival. However, in other tumor types, TATE as a prognostic marker for survival has been a controversial issue. This may be because of differences in methods of counting TATE as well as heterogeneity of material.

Previous studies have demonstrated that cytokines such as IL-2, IL-4 could recruit eosinophils and lead to eosinophilia and enhanced eosinophil activation, thereby exert potent anti-tumor immune responses [41, 46]. Thus, based on our present result that TATE improving survival in human solid tumors identified in this study and the function of IL-2 and IL-4 stated above, we harbor the idea that clinical application of biological response modifiers (BRM) such as carrier-assisted recombined human IL-2 /or IL-4 may have the potential to treat human solid tumors.

Quite a few limitations should be noted from this study. First, morphometric analyses for TATE adopted in included researches were not exactly consistent. In addition, researches with negative results might not be published, which might result in potential publication bias.

## Conclusions

TATE promotes survival in solid tumors especially in CRC and EC, suggesting that it is a valuable prognostic biomarker and clinical application of biological response modifiers or agonists promoting TATE may be a novel therapeutic strategy for patients.

## Supplementary information

**Additional file 1: Figure S1.** Flow chart diagram of study selection. **Figure S2.** Plots describing the influence of individual studies on the overall HRs for OS (**A**) and DFS (**B**) in human cancers. **Figure S3.** Funnel plots displayed the potential publication bias between TATE and OS (**A**) or DFS (**B**) in patients. **Table S1.** Characteristics of the included studies for OR analysis of clinicopathological features.

## Data Availability

The datasets supporting the conclusions of this article are included within the article.

## Abbreviations

TATE: Tumor-associated tissue eosinophilia
OS: Overall survival
DFS: Disease-free survival
HR: Hazard ratio
OR: Odds ratio
Cl: Confidence interval
TNM: Tumor, node, metastasis
OC: Oral cancer
CRC: Colorectal cancer
EC: Esophageal carcinoma
NR: Not reported
TME: Tumor microenvironment
BRM: Biological response modifier
