# Supplementary material for: Tumor-associated tissue eosinophilia predicts favorable clinical outcome in solid tumors: a meta-analysis
Source: BMC Cancer. 2020 May 20;20:454. doi: 10.1186/s12885-020-06966-3 (PMC7240929; doi:10.1186/s12885-020-06966-3)
Supplement: Supplementary file 1 — Additional file 1: Figure S1. Flow chart diagram of study selection. Figure S2. Plots describing the influence of individual studies on the overall HRs for OS (A) and DFS (B) in human cancers. Figure S3. Funnel plots displayed the potential publication bias between TATE and OS (A) or DFS (B) in patients. Table S1. Characteristics of the included studies for OR analysis of clinicopathological features. [file 12885_2020_6966_MOESM1_ESM.pdf]

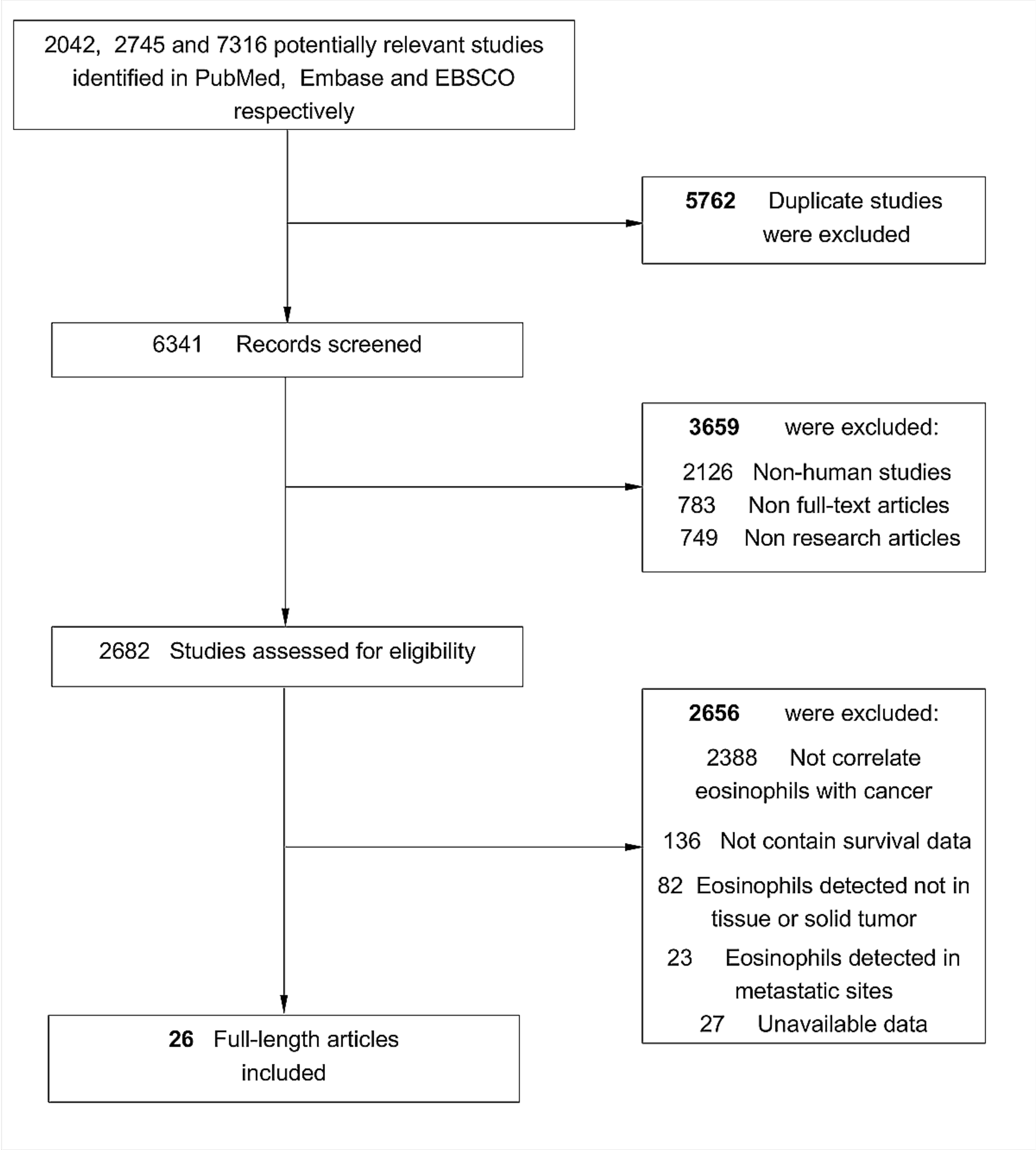

Fig. S1. Flow chart diagram of study selection.

A. OS

B. DFS

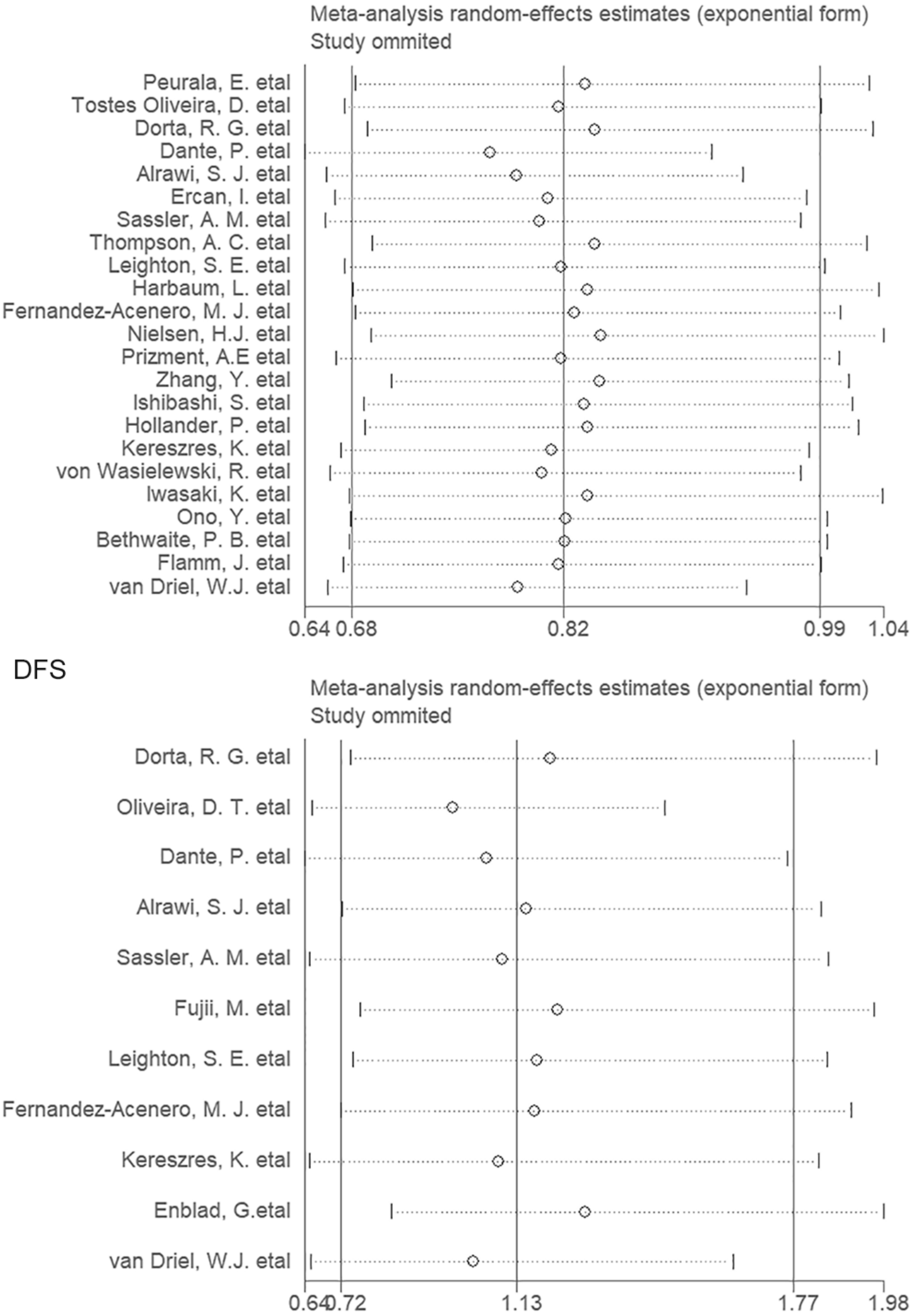

Fig. S2. Plots describing the influence of individual studies on the overall HRs for OS (**A**) and DFS (**B**) in human cancers.

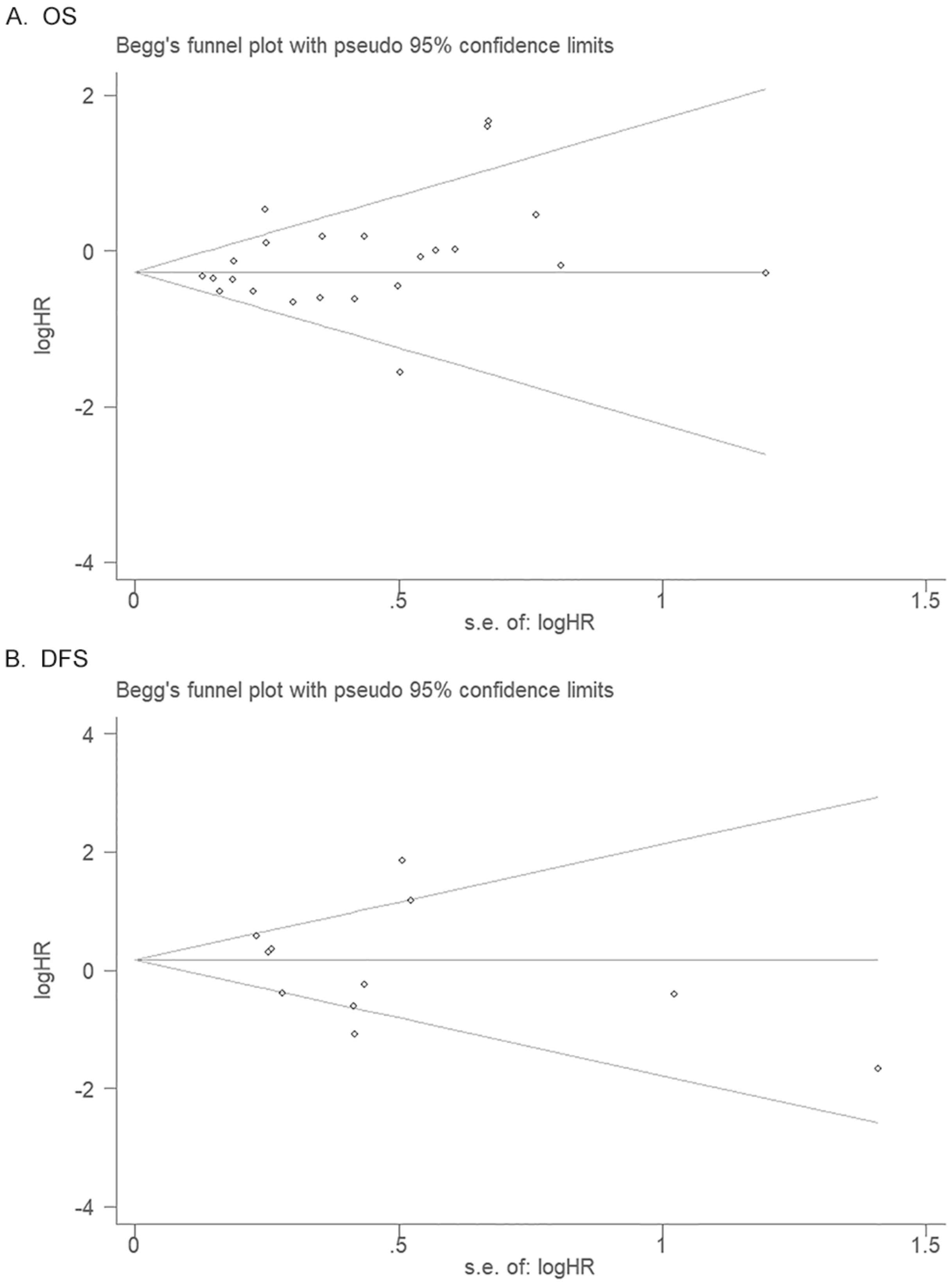

Fig. S3. Funnel plots displayed the potential publication bias between TATE and OS (**A**) or DFS (**B**) in patients.

Table S1. Characteristics of the included studies for OR analysis of clinicopathological features.

Study	Year	Tumor type	No. of Patients	TATE: Present / absent	Lymph node metastasis (Yes / No)	Tumor stage	Ⅰ+Ⅱ / Ⅲ+Ⅳ	Lymphatic invasion (Yes / No)	Vascular invasion (Yes / No)
Tostes Oliveira, D. etal [19]	2009	Oral cancer	43	21/22	P:(6/15); A:(5/17)	Ⅰ - Ⅳ	P:(6/15); A:(9/13)	NR	NR
Leighton, S. E. etal [24]	1996	Nasopharyngeal carcinoma	96	65/31	P:(49/16); A:(24/7)	NR	NR	NR	NR
Harbaum, L. etal [25]	2015	Colorectal cancer	381	101/280	P:(33/68); A:(135/145)	Ⅳ	P:(66/35); A:(135/145)	P:(25/76); A:(101/179)	P:(19/82)； A:(68/212)
Fernandez-Acenero, M. J. etal [26]	2000	Colorectal cancer	126	29/97	P:(27/2); A:(94/3)	Duke's A-C	NR	NR	P:(70/9); A:(113/21)
Zhang, Y. etal [29]	2014	Esophageal carcinoma	36	18/18	P:(9/9); A:(13/5)	Ⅰ - Ⅳ	P:(11/7)； A:(13/5)	NR	NR
Oliveira, D. T. etal [16]	2012	Oral cancer	71	35/36	NR	Ⅰ - Ⅱ	NR	P:(9/26); A:(14/22)	P:(30/5)；A:(31/5)

T: primary tumor; N: lymph node; M: metastasis; NR: not reported. P: present; A: absent.
